# Predictors of poor mental health among nurses during COVID‐19 pandemic

**DOI:** 10.1002/nop2.697

**Published:** 2020-11-20

**Authors:** Son Chae Kim, Carlota Quiban, Christine Sloan, Anna Montejano

**Affiliations:** ^1^ School of Nursing Point Loma Nazarene University San Diego CA USA

**Keywords:** anxiety, coping mechanisms, COVID‐19, depression, nurses, pandemic, stress

## Abstract

**Aims:**

To examine the impact of various factors affecting nurses' mental health during the COVID‐19 pandemic.

**Design:**

An online cross‐sectional study.

**Methods:**

Registered nurses who graduated from a nursing school in Southern California, USA, participated in the study from 20 April–10 May 2020 (*N* = 320). Kendall's tau correlations and multivariate logistic regression procedures were performed with stress, anxiety and depression as outcome variables.

**Results:**

Most nurses reported moderate/high stress (80.1%), while 43% and 26% reported moderate/severe anxiety and depression, respectively. COVID‐19 patient care was positively associated with moderate/severe high stress (OR = 2.25; *p* = .012) and moderate/severe anxiety (OR = 3.04; *p* < .001), whereas quarantine was associated with moderate/severe depression (OR = 2.68; *p* < .001). High levels of family functioning, resilience and spirituality predicted two‐ to sixfold lower odds of moderate/severe stress, anxiety or depression. High resilience, spirituality and family functioning appear to be good coping mechanisms for nurses against stress, anxiety and depression during the pandemic.

## 1 INTRODUCTION

The global COVID‐19 pandemic has created a massive public health crisis and numerous challenges for healthcare workers. This infection caused by SARS‐CoV‐2 virus is the worst pandemic since the Spanish flu a century ago with its rapid person‐to‐person spread that has overwhelmed many healthcare systems (Mareiniss, 2020; Tsamakis et al., 2020). Frontline healthcare workers experience high exposure to the virus. In Italy, some 10,000 healthcare workers were infected as of early April 2020, requiring quarantine or self‐isolation of many essential workers (Chersich et al., 2020). Increased work intensity, absence of effective treatment or vaccine, high infectivity and the fear of infecting loved ones have had significant negative impacts on the mental health of the healthcare workers as they care for COVID‐19 patients (Kisely et al., 2020). It is essential to understand various factors that influence the psychological well‐being of frontline healthcare workers during this extraordinary pandemic, which could help in designing specific interventions to minimize negative sequelae (Arden & Chilcot, 2020; Hyun et al., 2020).

## 2 BACKGROUND

Psychological distress and poor mental health among frontline healthcare workers during the COVID‐19 pandemic have been reported. A cross‐sectional survey among 1,257 healthcare workers taking care of COVID‐19 patients in China showed that about half of the participants had symptoms of anxiety and depression (Lai et al., 2020). According to the report, nurses had a significantly higher incidence of severe depression compared with physicians. Healthcare workers in “designated” COVID‐19 hospitals had two‐ to threefold higher odds of distress, anxiety and depression compared with those in non‐designated hospitals. Similar study results were also reported among frontline healthcare workers caring for COVID‐19 patients in China (Mo et al., 2020; Wu & Wei, 2020).

In addition to the immediate ongoing impact, sustained longer‐term effects have been observed among healthcare workers following the earlier SARS outbreak in 2003. For example, 1–2 years after the SARS outbreak in Canada, frontline healthcare workers were still having higher burnout, psychological distress and post‐traumatic stress disorder compared with those who did not care for SARS patients (Maunder et al., 2006). Furthermore, the same study reported an increase in smoking and drinking among those who cared for SARS patients. Similar results from Hong Kong were also noted 1 year after the SARS outbreak (McAlonan et al., 2007). Correlation between caring for SARS patients and the mental health impact, such as emotional exhaustion, state anger and avoidance behaviour, was found among nurses 1 year after the SARS outbreak (Marjanovic et al., 2007). In this study, the nurses' perception of inadequate organizational support was a significant predictor of poor mental health.

These short‐ and longer‐term adverse effects of epidemics on the frontline healthcare workers' mental health have called attention to organizational needs to provide comprehensive support for their employees (Hyun et al., 2020). A systematic review of 22 studies on the SARS outbreak showed that supportive environment, training and overall organizational preparedness have protective effects on the mental health of the healthcare workers (Brooks et al., 2018). Reduced anxiety and depression symptoms, as well as better sleep quality, were observed among nurses after the implementation of an organizational preparedness programme for potential epidemics (Chen et al., 2006). Furthermore, having counselling and psychological support from hospitals during the SARS outbreak reduced psychological distress by 50% among frontline healthcare workers (Tam et al., 2004). Having a better understanding, as well as infection control support from the hospitals, was associated with higher resilience among staff who were in contact with COVID‐19 patients (Huang et al., 2020).

Recently, more attention has been paid to resilience training programmes at the organizational level to improve the long‐term mental well‐being and stress management of frontline healthcare workers. Resilience refers to the individual's ability to “bounce‐back” in the face of adversity, which reduces perceived stress and helps the person to adapt constructively (Joyce et al.,  2019). For the implementation of a cost‐effective training for pandemic preparedness, computer‐assisted resilience training programmes of various lengths were tested in a randomized controlled trial (Maunder et al., 2010). The shortest training duration of 1.75 hr was significantly superior to training durations of 3.0 or 4.5 hr for the pandemic self‐efficacy outcome. Interestingly, none of the coping‐skill subscales, including problem‐solving, seeking support and escape avoidance, were improved by resilience training. More recently, 6 weeks of Internet‐based resilience training for firefighters in a cluster randomized controlled study showed improvement in adaptive resilience immediately at the end of the training and 6 months later (Joyce et al., 2019).

Therefore, it appears that the cost‐effective training may be useful for improving resilience, which could further improve psychological well‐being for frontline healthcare workers during epidemics. In addition to resilience training and infection control measures, social support is also a known predictor of mental health during infectious outbreaks. Inadequate family support was associated with nurses' burnout, anxiety and depression (Chen et al., 2006; Kim & Choi, 2016). A more recent qualitative study of frontline healthcare workers caring for COVID‐19 patients also showed beneficial effects of social support (Liu et al., 2020).

Although recent studies have described poor mental health among healthcare workers during the COVID‐19 pandemic, the impact of various factors mitigating perceived stress, anxiety or depression has not been thoroughly examined (Lai et al., 2020; Mo et al., 2020; Wu & Wei, 2020). Furthermore, little is known about the association between various coping mechanisms and nurses' mental health during the COVID‐19 pandemic. Such knowledge could help organizations identify the most beneficial interventions to improve and sustain the psychological well‐being of frontline nurses during the COVID‐19 crisis.

### 2.1 Aims

The specific aims of this study were to compare the nurses' mental health status before and during the COVID‐19 pandemic and to examine the impact of various factors affecting their mental health.

## 3 THE STUDY

### 3.1 Study design

A web‐based cross‐sectional study was conducted using an online survey platform, Qualtrics^XM^ (Provo, UT, USA), from 20 April–10 May 2020.

### 3.2 Participants

All registered nurses who graduated from the nursing school at a private, 4‐year liberal arts university in Southern California, USA, were invited to participate in the study. The nursing school offers a Bachelor of Science in Nursing (BSN) degree and graduate degrees, such as Master of Science in Nursing (MSN) and Doctorate in Nursing Practice (DNP). The number of alumni with available contact information was 1,397. The inclusion criteria for the study were registered nurses from either bachelor or graduate programme and active in any nursing field. The alumni who reported themselves as retired were excluded from the analysis.

### 3.3 Instruments

The study survey included valid and reliable instruments that measure stress, resilience, family functioning, spirituality, anxiety and depression. Demographic data such as age, gender, ethnicity, educational background, work setting and hours, years of RN experience and COVID‐19 patient care, as well as quarantine and self‐isolation experience, were also included. Participants were asked to recall their symptoms of stress, anxiety and depression before and during the pandemic.

The 10‐item Perceived Stress Scale (PSS) assesses the participants' perceived psychological stress by rating their feelings and thoughts during the past month (Cohen et al., 1983). It consists of two subscales, including 6‐item positive factor asking the ability to manage the stressors and 4‐item negative factor. With the response options on a 5‐point Likert scale ranging from 0 (never)–4 (very often), the summation scores range from 0–40 with a higher score indicating a higher level of stress. The scores from 0–13 indicate low stress, whereas scores from 14–26 and 27–40 indicate moderate and high levels of stress, respectively. Cronbach's alpha for the PSS was reported as 0.83 (Leung et al., 2010). In our study, Cronbach's alpha was 0.87.

The Connor‐Davidson Resilience Scale (CD‐RISC)‐10 consists of 10 items rating resilience and ability to cope with challenges during the past month (Connor & Davidson, 2003). The items rate the respondent's ability to adapt to changes, think clearly under pressure and see the humorous side of things when faced with problems, on a 5‐point Likert scale ranging from 0 (not true at all)–4 (true all the time). The summation scores range from 0–40, with a higher score indicating a high level of resilience. The internal consistency reliability was reported as Cronbach's alpha of 0.92 (Guo et al., 2018). In our study, Cronbach's alpha of the CD‐RISC‐10 was 0.86.

The Family APGAR measures five indicators of family functioning, such as Adaptation, Partnership, Growth, Affection and Resolve on a three‐point Likert scale ranging from 0 (hardly ever)–2 (almost always) (Smilkstein et al., 1982). Scores of 8–10 indicate highly functional families, whereas scores of 4–7 and 0–3 indicate moderately dysfunctional and dysfunctional families, respectively. The internal consistency was reported as inter‐item correlation coefficients ranging from 0.63–0.71 (Gardner et al., 2001).

The 12‐item Spirituality Support Scale measures the perceived support received from higher powers in participants' faith experience on a 4‐point Likert scale, ranging from 1 (strongly disagree)–4 (strongly agree). The higher score indicates higher spirituality support, and Cronbach's alpha was reported as 0.97 (Ai et al., 2005). Likewise, Cronbach's alpha of the Spirituality Support Scale in our study was 0.97.

The General Anxiety Disorder‐7 (GAD‐7) and Patient Health Questionnaire‐9 (PHQ‐9) are widely used tools that assess the symptom severity of anxiety and depression, respectively (Spitzer et al., 1999, 2006). Each question asks how often the participants have been bothered with the indicators over the past 2 weeks on a 4‐point Likert scale, ranging from 0 (not at all)–3 (nearly every day). With the GAD‐7 scores, the severity criteria of anxiety symptoms are as follows: none/minimal (0–4), mild (5–9), moderate (10–14) and severe (15–21). The severity of depression symptoms using PHQ‐9 scores is as follows: none/minimal (0–4), mild (5–9), moderate (10–14), moderately severe (15–19) and severe (20–27). Scores of 10 or higher in the GAD‐7 and the PHQ‐9 indicate moderate/severe symptoms that require further evaluation. The sensitivity and specificity of the GAD‐7 in screening social anxiety disorder were 72% and 80%, respectively (Spitzer et al., 2006). Similarly, the sensitivity and specificity of the PHQ‐9 in screening major depressive disorder were 88% and 88%, respectively.

### 3.4 Data collection procedures

The initial recruitment email containing the hyperlink to the online survey was sent to all nursing alumni with available contact information from the university alumni association. A second reminder email was sent after the first week, resulting in data collection from 20 April–10 May 2020. A $20 gift card was sent to 10 randomly selected winners after the closure of the study.

### 3.5 Data analysis

Descriptive statistics of mean, median, interquartile range (IQR), frequency and percentage summarized the sample characteristics and key variables. Wilcoxon signed‐rank tests were performed to compare the scores of stress, anxiety and depression before versus during the pandemic. To examine the factors affecting mental health, the scores of outcome variables were recoded as dichotomous variables according to the criteria of the symptom severity. Poor mental health was defined as moderate/high stress, as well as moderate/severe anxiety and depression. For example, total PSS scores ≥ 14 were recoded as moderate/high stress (=1), whereas scores < 14 were recoded as low stress (=0). Similarly, total GAD‐7 and PHQ‐9 scores were recoded as dichotomous variables, with scores ≥ 10 as “1” (moderate/severe anxiety or moderate/severe depression) and scores < 10 as “0” (none or mild). Coping mechanisms were also recoded as dichotomous variables. The CD‐RISC‐10 scores were recoded as “1” (high resilience; ≥ median score of 30) and “0” (low resilience; scores < 30). The Spirituality Support Scale scores were recoded as high spirituality (=1; ≥ median score of 39) versus low spirituality (=0; scores < 39). The family APGAR score ≥ median score of 10 was recoded as “1” (high family functioning) and the rest as “0” (low family functioning).

Bivariate Kendall's tau correlation procedures were first performed to examine the correlations between poor mental health and various explanatory variables, including demographics and coping mechanisms. Subsequently, multivariate logistic regression procedures were performed by entering all statistically significant explanatory variables from the Kendall tau tests to determine the predictors of poor mental health. The SPSS version 26.0 (IBM Corporation, Armonk, NY) was used for data analysis, and the level of significance was set at *α* < 0.05.

### 3.6 Ethical considerations

This study was reviewed and approved by a university Institutional Review Board. Signed informed consent was waived due to the minimal risks involved in this online survey. Potential participants were reminded that participation in the study was entirely voluntary and their choice to participate would not affect their relationship with the nursing school. Completion of the online survey indicated their consent to the study. This study was conducted in accordance with the Declaration of Helsinki.

## 4 RESULTS

### 4.1 Sample characteristics

Of the 653 alumni who received and opened the invitation email, 324 completed the survey (49.6% completion rate). Four retirees were excluded, resulting in 320 eligible participants included in data analysis. The average age was 33 years, and the most were female (94.4%) (Table 1). The average RN experience was 10 years. Most were white (73.1%), working at acute care hospitals (75.9%), working full time (82.2%) and had bachelor's degrees (75.3%). Approximately half took care of COVID‐19 patients (48.8%) and experienced quarantine or self‐isolation during the COVID‐19 pandemic (45.4%).

**Table 1:** Sample characteristics (*N* = 320)

	*N* (%)
Age, mean (range), years	33 (21–67)
Gender	
Female	302 (94.4)
Male	18 (5.6)
Ethnicity	
White	234 (73.1)
Hispanic	33 (10.3)
Asian/Pacific Islanders	33 (10.3)
African American	5 (1.6)
Other	15 (4.7)
Educational background	
Bachelor	244 (75.3)
Master's	65 (20.3)
Doctoral	13 (4.1)
Work setting	
Acute care hospital	243 (75.9)
Primary care clinic	19 (5.9)
Academic setting	18 (5.6)
Skilled nursing facility	2 (0.6)
Other	48 (15.0)
Working hours per week	
≥30 hr	263 (82.2)
20–29 hr	27 (8.4)
<20 hr	30 (9.4)
Years of RN experience, mean (range)	10.1 (0–44)
COVID−19 patient care	158 (48.8)
Quarantine or self‐isolation experience	147 (45.4)

Note

Values are expressed as *N* (%) unless otherwise indicated. Percentage may not add to 100% because of missing data or rounding.

### 4.2 Mental health before and during the pandemic and coping mechanisms

**Figure 1:**
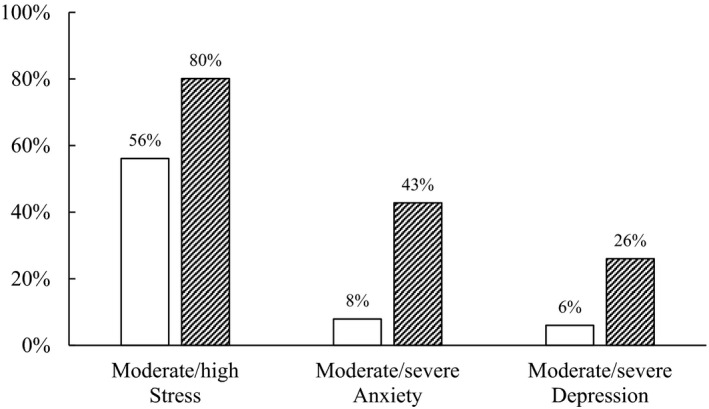
Nurses with poor mental health before and during COVID‐19 pandemic (*N* = 320). White bars and crosshatch bars represent poor mental health before and during COVID‐19 pandemic, respectively. Moderate/high stress = Perceived Stress Scale scores ≥ 14; Moderate/severe anxiety = GAD‐7 scores ≥ 10; Moderate/severe depression = PHQ‐9 scores ≥ 10

Before the COVID‐19 pandemic, the median (IQR) scores of PSS for stress, GAD‐7 for anxiety and PHQ‐9 for depression were 14 (11, 17), 4 (1, 6.5) and 2 (1, 5), respectively. These median scores were increased significantly during the COVID‐19 pandemic: 18 (15, 22), 8 (4, 13) and 6 (3, 10) for stress, anxiety and depression, respectively (*p* < .001). During this pandemic, most nurses reported moderate/high level of perceived stress (80%), while 43% and 26% reported moderate/severe levels of anxiety and depression, respectively (Figure 1). The median (IQR) scores of resilience, spirituality and family functioning were 30 (28, 34), 39 (34, 46) and 10 (8, 10), respectively.

### 4.3 Correlations between poor mental health and coping mechanisms

The results of bivariate Kendall's tau correlations between the poor mental health and coping mechanisms and demographic factors during the pandemic are shown in Table 2. The findings showed that moderate/high stress was negatively correlated with age, years of RN experience, high resilience, high family functioning and high spirituality. In contrast, female gender and caring for COVID‐19 patients were positively correlated with moderate/high stress. For moderate/severe anxiety and depression, there were negative correlations with age, years of RN experience, high resilience, high family functioning and high spirituality. Caring for COVID‐19 patients was positively correlated with moderate/severe anxiety. However, quarantine or self‐isolation experience was positively correlated only with moderate/severe depression.

**Table 2:** Correlations of demographic factors/coping skills with poor mental health during COVID‐19 pandemic (*N* = 320)

	Moderate/high stress	Moderate/severe anxiety	Moderate/severe depression
Age	−0.25***	−0.15**	−0.13*
Female	0.15**	0.07	0.05
Years of RN experience	−0.22***	−0.13*	−0.15*
COVID‐19 patient care	0.15**	0.23***	0.08
Quarantine or self‐isolation experience	0.02	0.06	0.18**
High resilience^a^	−0.30***	−0.26***	−0.19**
High family functioning^b^	−0.21***	−0.20**	−0.22***
High spirituality^c^	−0.18**	−0.20***	−0.23***

Note

Correlation coefficients by Kendall's Tau test. Moderate/high stress = Perceived Stress Scale scores ≥ 14; Moderate/severe anxiety = GAD‐7 scores ≥ 10; Moderate/severe depression = PHQ‐9 scores ≥ 10.

^a^ CD‐RISC‐10 scores ≥ 30.

^b^ Family APGAR scores ≥ 10.

^c^ Spirituality Support Scale scores ≥ 39.

* *p* < .05.

** *p* < .01.

*** *p* < .001.

### 4.4 Predictors of poor mental health

Table 3 showed the results of multivariate logistic regression procedures by entering the statistically significant variables from bivariate Kendall's tau tests. For moderate/high stress, age (odds ratio [OR] = 0.97; 95% confidence interval [CI] [0.94, 0.99]; *p* = .007), high resilience (OR = 0.18; 95% CI [0.08, 0.41]; *p* < .001) and high family functioning (OR = 0.37; 95% CI [0.19, 0.73]; *p* = .004) were significant negative predictors. In contrast, caring for COVID‐19 patients was a significant positive predictor for moderate/high stress (OR = 2.25; 95% CI [1.12, 4.24]; *p* = .012).

**Table 3:** Predictors of poor mental health during COVID‐19 pandemic (*N* = 320)

	OR	95% CI	*p*‐value
Moderate/high stress			
COVID‐19 patient care	2.25	1.12–4.24	.012
Age	0.97	0.94–0.99	.007
High resilience^a^	0.18	0.08–0.41	<.001
High family functioning^b^	0.37	0.19–0.73	.004
Moderate/severe anxiety			
COVID‐19 patient care	3.04	1.86–4.96	<.001
High resilience^a^	0.34	0.21–0.56	<.001
High family functioning^b^	0.52	0.32–0.84	.008
Moderate/severe depression			
Quarantine or self‐isolation experience	2.68	1.55–4.63	<.001
High spirituality^c^	0.38	0.21–0.66	.001
High family functioning^b^	0.40	0.23–0.69	.001

Note

Moderate/high stress = Perceived Stress Scale scores ≥ 14; Moderate/severe anxiety = GAD‐7 scores ≥ 10; Moderate/severe depression = PHQ‐9 scores ≥ 10.

Abbreviations: CI, confidence interval by multivariate logistic regressions; OR, odds ratio.

^a^ CD‐RISC‐10 scores ≥ 30.

^b^ Family APGAR scores ≥ 10.

^c^ Spirituality Support Scale scores ≥ 39.

For moderate/severe anxiety, caring for COVID‐19 patients (OR = 3.04; 95% CI [1.86, 4.96]; *p* < .001) was a positive predictor, whereas high resilience (OR = 0.34; 95% CI [0.21, 0.56]; *p* < .001) and high family functioning (OR = 0.52; 95% CI [0.32, 0.84]; *p* = .008) were significant negative predictors. For moderate/severe depression, quarantine or self‐isolation was a significant positive predictor (OR = 2.68; 95% CI [1.55, 4.63]; *p* < .001). In contrast, high spirituality (OR = 0.38; 95% CI [0.21, 0.66]; *p* = .001) and high family functioning (OR = 0.40; 95% CI [0.23, 0.69]; *p* = .001) were significant negative predictors.

## 5 DISCUSSION

This study shows that nurses are experiencing poor mental health during the COVID‐19 pandemic, with most nurses reporting moderate/high levels of stress, whereas 43% and 26% reporting moderate/severe anxiety and depression symptoms, respectively. These symptoms are significantly higher than those estimated by the same nurses before the pandemic. It was not surprising that poor mental health was positively associated with COVID‐19 patient care and quarantine/self‐isolation experience. However, the effect size of threefold higher odds of anxiety related to COVID‐19 patient care in a multivariate analysis was unexpected. This large effect size may be due to the fear of the new pandemic sweeping across the globe, fear about passing the virus to family members, and the current lack of treatment or vaccine (Chersich et al., 2020; Kisely et al., 2020). Likewise, quarantine/social isolation experience was associated with almost threefold higher odds of moderate/severe depression. These results are consistent with previous reports. For example, higher levels of anxiety and depression were found among frontline healthcare workers in a hospital designated for COVID‐19 in China compared with those in a non‐designated hospital (Lai et al., 2020; Wu & Wei, 2020). During an earlier SARS outbreak in Canada, frequent symptoms of PTSD and depression were reported among the quarantined individuals (Hawryluck et al., 2004).

In bivariate correlational analyses, demographic variables such as age and years of RN experiences were negatively correlated with poor mental health. This indicates that older nurses with more extensive RN experience have lower stress, anxiety and depression. Similarly, in multivariate analyses, age was a negative demographic predictor of moderate/high stress. The odds ratio of 0.97 indicates a 3% decrease in the odds of moderate/high stress for each year of increase in age. This decrease in perceived stress associated with age is most likely due to higher maturity and work experience, as nurses get older. Others have found similar results that nurses who are younger with less clinical experience tend to have higher levels of stress (Cheung & Yip, 2015).

During the time of global crisis like the COVID‐19 pandemic, nurses must possess coping mechanisms and have sources of social support to manage the stress while providing quality care for patients. In this study, nurses with high levels of resilience, spirituality and family functioning had two‐ to sixfold lower odds of poor mental health. For example, nurses with high resilience had odds ratio of only 0.18 for the moderate/high stress, indicating a 5.6‐fold lower likelihood of perceived stress. The resilience scale used in this study assesses whether the individual can adapt to changes, think clearly under pressure and believe that coping with stress can make one stronger (Connor & Davidson, 2003). These examples of resilience represent characteristics of personal tenacity and competence in adversity, as well as adaptability and a sense of being in control during a crisis. This study finding is also consistent with previous reports that resilience protects nurses from work‐related stress and is a negative predictor of psychological distress (Guo et al., 2019; Zou et al., 2016).

Although there has been much focus on resilience as a coping mechanism because of the potential for training to improve resilience, we have found that other potentially modifiable factors may be important as well for nurses' mental health. In our study, we have found that having high family functioning was an independent predictor of all three poor mental health outcomes. High level of family functioning reduced the odds of stress, anxiety and depression by two‐to threefold. Apparently, having satisfactory family support and affection during difficult times has a broad positive impact across multiple dimensions of psychological well‐being, including stress, anxiety and depression. A similar finding was reported after the 2008 Wenchuan earthquake (Cao et al., 2013). In this study from China, positive family functioning was associated with better health status even 18 months after the disaster.

In addition, we have found that nurses with high spirituality had almost a threefold lower odds of moderate/high depression. Among the nurses studied, it appears that support from a religious association, inner strength from spiritual faith, and peace and contentment from faith helps protect against depression. A previous study also reported that spiritual support was positively associated with long‐term mental health among residents who experienced multiple disasters of hurricanes and oil spills (Cherry et al., 2018).

We have assessed the role of resilience, spirituality and family functioning as multidimensional aspects of coping during the ongoing COVID‐19 pandemic. Each of these dimensions appears to be an independent predictor of mental health among nurses. These are potentially modifiable through counselling, training, role modelling and other means (Cherry et al., 2018; Joyce et al., 2019). Recognition of the importance of these coping mechanisms can help nurses manage their psychological distress during significant crisis and prevent long‐term adverse effects on their mental health (Chersich et al., 2020).

### 5.1 Limitations

There are several limitations to this study. First, the associations between coping mechanisms and mental health should not be taken as a causal relationship in this cross‐sectional study. Second, a convenience sampling of alumni from one nursing school in this study may limit the generalizability of the findings. Third, self‐reporting of the mental health symptoms may have introduced bias through over‐ or under‐estimation of the respondents' symptoms. Fourth, among the invited alumni, those with poor mental health may have self‐selected to complete the survey, resulting in potential over‐estimation of the symptoms. Finally, the mental health status of respondents before the pandemic was collected retrospectively, which may have resulted in recall bias. Future studies are needed to understand the long‐term mental health impacts of the global COVID‐19 pandemic among nurses.

## 6 CONCLUSION

This study showed a high proportion of poor mental health among nurses, especially those who provided care to COVID‐19 patients or experienced quarantine/social isolation. High resilience, spirituality and family functioning were significant predictors of mental health during the COVID‐19 pandemic. Strengthening these multidimensional coping mechanisms among nurses may enhance their psychological well‐being during times of pandemic and also reduce long‐term negative consequences. Nurses with good mental health will be able to provide high quality and safe patient care.

## CONFLICT OF INTEREST

None.

## AUTHOR CONTRIBUTIONS

SCK, CQ, CS, AM: Substantial contributions to conception and design, or acquisition of data, or analysis and interpretation of data; drafting of the manuscript or revising it critically for important intellectual content; final approval of the version to be published; accountable for all aspects of the work in ensuring that questions related to the accuracy or integrity of any part of the work are appropriately investigated and resolved.

## Data Availability

The data that support the findings of this study are available from the corresponding author upon reasonable request.
